# Adjunctive qiliqiangxin capsule for heart failure in predominantly middle-aged and older adults: a systematic review and meta-analysis of randomized controlled trials

**DOI:** 10.3389/fcvm.2026.1881321

**Published:** 2026-08-12

**Authors:** Shun Han, Xingxing Li, Xue Gao, Xuebin Chen

**Affiliations:** 1 Shaanxi University of Chinese Medicine, Xianyang, China; 2 Department of Cardiology, The Affiliated Hospital of Shaanxi University of Chinese Medicine, Xianyang, China

**Keywords:** cardiac function, heart failure, middle-aged and older adults, qiliqiangxin capsule, systematic review and meta-analysis

## Abstract

**Background:**

Heart failure (HF) is highly prevalent among middle-aged and older adults and remains associated with substantial residual morbidity and mortality despite guideline-guided treatment. Previous meta-analyses of Qiliqiangxin Capsule (QLQX) have mainly focused on general HF populations or patients with HF with reduced ejection fraction (HFrEF), with limited attention to age-related clinical applicability. Therefore, this study evaluated the adjunctive effects of QLQX in predominantly middle-aged and older study populations with HF.

**Objective:**

To evaluate the efficacy of QLQX combined with conventional therapies (CTs) on cardiac function in middle-aged and older HF patients.

**Methods:**

Seven databases were searched from inception to January 2026 for RCTs of QLQX. Two reviewers independently screened studies and extracted data. The risk of bias and the certainty of the evidence were assessed using the Cochrane risk-of-bias tool and GRADE. Meta-analysis used random-effects models for continuous outcomes and fixed-effects models for dichotomous outcomes when heterogeneity was low. Subgroup and sensitivity analyses explored heterogeneity and tested robustness.

**Results:**

Eleven RCTs involving 4,642 participants were included. Compared with CTs alone or placebo plus CTs, QLQX combined with CTs was associated with higher LVEF across 10 trials involving 1,532 participants [MD = 5.77, 95% CI (4.32, 7.22)] and lower NT-proBNP levels [MD = −312.98 pg/mL, 95% CI (−418.31, −207.64)]. The pooled estimate also favored QLQX for LVEDD [MD = −3.50, 95% CI (−4.65, −2.35)]. HF hospitalization or rehospitalization was reported in two trials, and the pooled cumulative risk estimate favored QLQX [RR = 0.80, 95% CI (0.69, 0.93)]. However, these findings should be interpreted with caution because substantial heterogeneity was observed across several outcomes, follow-up durations varied across studies, and evidence for hard clinical endpoints remained limited, with outcome-specific certainty ranging from moderate to very low.

**Conclusion:**

QLQX combined with CTs may be associated with short- to medium-term improvements in surrogate measures of cardiac function and functional status in predominantly middle-aged and older study populations with HF. However, evidence regarding hard clinical endpoints remains limited, and large-scale, multicenter, high-quality, double-blind RCTs are needed to clarify its long-term efficacy and prognostic value.

**Systematic Review Registration:**

https://www.crd.york.ac.uk/PROSPERO/view/CRD420261295659, identifier CRD420261295659.

## 1 Introduction

Heart failure (HF) is a complex clinical syndrome associated with high morbidity, high mortality, and frequent hospital readmissions. Epidemiological evidence indicates that the global prevalence of HF continues to rise, driven by population ageing, longer survival among patients with underlying cardiovascular disease, and the increasing burden of cardiometabolic risk factors. HF affects tens of millions of individuals worldwide and contributes substantially to disability and impaired quality of life (1). The rising prevalence of HF has resulted in a considerable economic burden, including both direct healthcare costs and indirect societal costs (2). These findings underscore the need for continued optimization of HF management.

Although the treatment strategy for HF has been continuously optimized, there are still many significant limitations. Patients with HF are often accompanied by a variety of complications, including chronic kidney disease, diabetes, and chronic lung disease (3). In clinical practice, such patients often show poor drug tolerance, difficulty in reaching the target treatment dose, and face a high residual risk of re-hospitalization due to recurrence. In addition, the efficacy of some new single-target therapies has tended to the platform stage (4), which makes the development of treatment plans that are both efficient and safe a very challenging task.

Traditional Chinese medicine (TCM) has a clinical application history of more than 2,000 years. As an important branch of supplementary medicine, traditional Chinese medicine has shown unique advantages in the prevention, treatment, and management of heart failure. Existing research evidence shows that traditional Chinese medicine can play a role through multi-target mechanisms, thus improving heart structure, inhibiting ventricular remodeling, and enhancing heart function; at the same time, traditional Chinese medicine also shows good safety and tolerance. The above findings further highlight the great potential of traditional Chinese medicine as an auxiliary therapy or alternative therapy in the primary and secondary prevention of HF (5, 6).

QLQX is a compound formulation comprising 11 traditional Chinese medicinal herbs, including *Astragali Radix, Ginseng Radix et Rhizoma, Aconiti Lateralis Radix Preparata, Salvia Miltiorrhiza Radix et Rhizoma, Descurainiae Semen et Lepidii Semen, Alismatis Rhizoma, Polygonati Odorati Rhizoma, Cinnamomi Ramulus, Carthami Flos, Periplocae Cortex,* and *Citri Reticulatae Pericarpium* (7). Chinese HF guidelines recommend it as an auxiliary therapy for HF (8). It is worth noting that when applied in combination with a standard HF treatment regimen, QLQX has been proposed to enhance heart function and improve clinical prognosis (9). Experimental studies show that QLQX can restore the inhibited peroxisome proliferator-activated receptor-α (PPAR-α) signaling pathway, downregulate the expression of glucose transporter 1 (GLUT1), and upregulate the expression of glucose transporter protein 4 (GLUT4), thus improving the metabolic flexibility of the heart. The synergy of its multiple active ingredients mediates the comprehensive heart protection effect (10).

In order to further clarify the efficacy of QLQX combined with CTs in the treatment of heart failure, a number of meta-analysis reports pointed out that QLQX combined with conventional therapy can significantly improve heart function compared with conventional therapy alone. It is worth noting that HF mainly involves middle-aged and older adults; this population usually has a poor prognosis, and the re-hospitalization rate and mortality rate are high. Most of the previous meta-analyses lack subgroup analysis for this specific population, thus limiting the ability to evaluate the effectiveness of differentiated treatment. To address this gap, the present study performed a meta-analysis of predominantly middle-aged and older HF populations with HF, systematically evaluating the effects of QLQX combined with CTs on cardiac function.

## 2 Methods

This systematic review and meta-analysis are carried out in accordance with the 2020 PRISMA Guide (11). For the detailed checklist, please see Supplementary File 1. This study program has been proactively registered in the PROSPERO database (registration number: CRD420261295659).

### 2.1 Search strategy

To identify eligible randomized controlled trials, we systematically searched seven databases from inception to January 2026: PubMed, Embase, Web of Science, The Cochrane Library, CNKI, WanFang, and CQVIP. The search strategy combined subject headings and free-text terms related to heart failure, Qiliqiangxin Capsules, and randomized controlled trials. The full search strategy is presented in Supplementary File 2. Grey literature, including dissertations and conference proceedings, was not systematically included because the methodological details and outcome data could not be adequately verified.

### 2.2 Selection criteria

**Inclusion Criteria:**

1. HF is diagnosed according to the standards used in the original trial; if possible, it is mapped to the contemporary ESC definition (12).
2. The study population is predominantly composed of middle-aged and older study populations, defined as having a mean or median age of ≥45 years (13), or as predominantly consisting of middle-aged and older adults, as explicitly described by the original authors (regardless of sex, nationality, or ethnicity).
3. The intervention group received QLQX in combination with CTs. CTs were defined as the background therapy used in each original trial, which could include: ACE inhibitors (ACEIs), angiotensin II receptor blockers (ARBs), angiotensin receptor neprilysin inhibitors (ARNIs), *β*-blockers, mineralocorticoid receptor antagonists (MRAs), diuretics, digoxin, or sodium glucose cotransporter 2 inhibitors (SGLT2i) when explicitly reported. While excluding other traditional Chinese medicine interventions. Importantly, CTs in this review did not necessarily align with contemporary guideline-directed therapy;
4. The control group received either standard treatment or a placebo, excluding other traditional Chinese medicine treatments;
5. At least one primary endpoint: LVEF, NT-proBNP, or LVEDD;
6. Randomized controlled trial design;

**Exclusion Criteria:**

1. Non-randomized controlled trials;
2. Acute heart failure patients;
3. Studies with a mean or median age below 45 years, those predominantly composed of younger adults, or those lacking sufficient age-related information to determine whether the population consists primarily of middle-aged and older adults will be excluded;
4. Participants with severe systemic diseases (e.g., end-stage renal disease, active malignancies, severe liver or pulmonary diseases, or pregnancy);
5. Studies employing traditional Chinese medicine treatments other than QLQX (e.g., acupuncture, massage, herbal injections);
6. Outcome measures that did not meet predefined criteria or could not be extracted;
7. Duplicate publications;
8. Publications without full text available;
9. Studies with incomplete or unavailable data.

### 2.3 Study selection and data extraction

All references were managed using EndNote X9, and duplicates were removed before screening. Two independent reviewers (Shun Han and Xingxing Li) conducted study selection in three stages. First, titles and abstracts were screened to exclude clearly irrelevant records. Second, the full texts of potentially eligible articles were assessed against the predefined inclusion and exclusion criteria. Third, data were independently extracted and cross-checked using standardized forms. Extracted data included study characteristics, participant characteristics, HF phenotype and diagnostic criteria, details of the intervention and comparator, background conventional therapy or guideline-directed medical therapy, treatment duration, follow-up and assessment time points, outcome definitions, and outcome measurement methods. For natriuretic peptide outcomes, we recorded whether natriuretic peptide outcomes were reported as NT-proBNP or BNP. Whether the data were presented as endpoint values, absolute changes from baseline, percent changes, or proportions achieving a prespecified reduction threshold, together with the sampling time point. For efficacy outcomes, we extracted data on NYHA grading-related clinical responses, LVEF, LVEDD, 6MWD, and clinical event outcomes, where available. Safety data, including adverse events and serious adverse events, were also extracted when reported. Any discrepancies were resolved through discussion, and a third reviewer was consulted when necessary.

### 2.4 Quality assessment and certainty of evidence

Given the limited methodological details reported in several included trials and to maintain comparability with previous evidence syntheses, two reviewers independently assessed the risk of bias in each included randomized controlled trial using the Cochrane Collaboration Risk of Bias tool (version RoB 1) (14). Each domain was assessed for low, high, or unclear risk of bias. Disagreements were resolved through discussion or adjudication by a third reviewer. Risk-of-bias graphs were generated using RevMan software (Version 5.4).

In addition, we used the GRADE method (15) to evaluate the certainty of the pre-set partial outcome evidence. Evidence evaluation covers five dimensions: bias risk, inconsistency, indirectness, inaccuracy, and publication bias. The overall certainty of the evidence is rated as high, medium, low, or extremely low. In the initial assessment, the evidence from the randomized controlled trial was assessed as highly certain; if serious doubts were found, it was downgraded. For evaluation differences, resolve them through discussion; if necessary, consult the third reviewer. The outcome summary table is generated by GRADEpro GDT software.

### 2.5 Outcomes and subgroups

The main endpoints of this meta-analysis include NT-proBNP, LVEF, and LVEDD. In addition, several secondary endpoints were evaluated, including 6MWD, NYHA grading-related clinical responses, and HF hospitalization or re-admission for HF. It is worth noting that, due to the significant differences in follow-up time among the included studies, short-term physiological responses and long-term clinical events cannot be directly used as comparable indicators of treatment efficacy. For example, short-term improvements in LVEF, LVEDD, or NT-proBNP may reflect hemodynamic regulation rather than durable structural or prognostic benefits, while long-term outcomes such as hospitalization reflect disease progression and structural remodeling. Therefore, these two types of indicators have not been incorporated into a unified interpretive framework.

For the continuous outcome index, if each research result is reported in a unified scale and unit, the mean difference (MD) is used as the effect indicator; If the outcome indicators with similar concepts are reported on different scales or units, the standardized mean difference (SMD) is used as the effect indicator. The two studies included reported NT-proBNP as the 12th week change relative to the baseline, and the data were presented in the form of a median (16, 17). For the continuity data reported in the form of a median with a discreteness indicator, this study uses the established statistical method to estimate its mean and standard deviation. Specifically, the average estimation formula is (Q1 + median + Q3)/3, and the standard deviation estimation formula is (Q3 − Q1)/1.35 (18). For the data before and after conversion, see the Supplementary File 5. A limited number of studies reported BNP levels; however, we chose NT-proBNP over BNP due to its superior stability and consistency in the clinical monitoring of HF. Among the studies included in the analysis, the majority reported NT-proBNP (16, 17, 19–23), which ensures the homogeneity of the combined data. Studies that only reported BNP were excluded from the pooled analysis to maintain consistency (24–26). Both the change scores and endpoint values reflect the same NT-proBNP outcome, measured on the same scale, which justifies the use of MDs in the pooled analysis. This study conducted subgroup analysis and sensitivity analysis to evaluate the robustness of the research results.

For dichotomous outcomes based on event counts, the summary estimate is calculated in the form of a risk ratio (RR) with a 95% confidence interval (CI). The reason why RR is chosen is that it can directly compare the probability of an event between treatment groups and provide clinically explainable effect estimates. In addition, when the incidence of outcome events is high, the odds ratio (OR) may have a significant deviation from the risk ratio, thus exaggerating the superficial magnitude of the therapeutic effect; therefore, RR is considered more applicable to outcomes with a relatively high incidence of events (27, 28).

For outcomes related to NYHA heart function grading, the included studies usually report the clinical efficacy category defined by the test side, rather than standardized NYHA grading changes. Therefore, in order to achieve quantitative synthesis, we re-code these categories into two classification outcomes: that is, “clinical improvement” and “not improved”. Subjects classified as “significantly effective” or “effective” are considered to have achieved clinical improvement; subjects classified as “ineffective” are considered to have not achieved clinical improvement. This recoded outcome indicator should be interpreted as the “clinical response” defined by the tester, rather than the unified improvement of the NYHA heart function grading.

For hard clinical outcomes, if consistent time-to-event data can be obtained, the hazard ratio (HR) is usually preferred as an effect indicator (29). However, HF hospitalization or re-admission for HF is included as a secondary outcome indicator, only two studies reported this outcome: one of them provided sufficient “time-to-event” information, including the Kaplan–Meier curve and the HR based on the Cox proportional hazards model (16); and the other only reported the cumulative number of events at the end of the follow-up period, and did not provide the HR value or the time of individual events (17). In view of the lack of “to the time of event” data and the Kaplan–Meier curve in the latter study, it is impossible to conduct an HR-based meta-analysis, and it is impossible to reconstruct the estimated value of “to the time of event”. In view of this, we used the RR to analyze HF hospitalization and re-hospitalization. The RR value is calculated according to the number of events in each group and the total sample size. When interpreting the RR value, it should be regarded as the cumulative risk of HF hospitalization at a specific follow-up point in each study, rather than as a comprehensive indicator to measure the effect of “to the time of event.”

To explore potential sources of heterogeneity, subgroup analyses were conducted. Prespecified variables included treatment duration (≤8 weeks vs. >8 weeks), age (middle-aged vs. elderly), and sample size.

In addition to the prespecified subgroup analyses, baseline LVEF-defined HF phenotype was examined in an exploratory study-level subgroup analysis to assess whether differences in HF phenotype contributed to between-study clinical heterogeneity. None of the included studies enrolled patients exclusively with HF with preserved ejection fraction (HFpEF). Accordingly, based on baseline LVEF, studies were stratified into HF with reduced ejection fraction (HFrEF, LVEF ≤40%) and HF with mildly reduced ejection fraction (HFmrEF, LVEF 41%–49%) (12).

Given that most studies did not report individual patient-level LVEF data, reclassification of heart failure phenotypes at the individual level was not feasible. Therefore, classification was performed at the study level using the original trial definitions and the mean baseline LVEF reported in each study.

### 2.6 Statistical analysis

Statistical analysis is carried out using Review Manager (RevMan, version 5.4) software. The summary effect is expressed by the effect estimate and its 95% CI, and a two-sided *P* value < 0.05 is considered statistically significant. All analyses are carried out according to the preset framework. For the two-classification outcome, only when statistical heterogeneity is low (I² < 25%) and clinical homogeneity is present across studies is the Mantel-Haenszel method under the fixed-effects model used to calculate the summary RR and its 95% CI (30). For the continuous outcome, given the clinical heterogeneity across studies in heart failure phenotype, background treatment, and follow-up duration, all analyses use random-effects models. Statistical heterogeneity is evaluated by Cochran's *Q* test and I² statistics (31). This study conducted a subgroup analysis to explore the potential source of heterogeneity and carried out a sensitivity analysis by excluding individual studies one by one to evaluate the robustness of the results. The publication bias is visually evaluated through the funnel diagram. Publication bias was assessed visually using funnel plots. For outcomes including at least 10 studies, funnel plot asymmetry was formally assessed using Egger's regression test in Stata 16.0. Formal asymmetry tests were not performed for outcomes with fewer than 10 studies because of limited power and the risk of misleading results in sparse meta-analyses (32).

## 3 Results

### 3.1 Study selection

**Figure 1:**
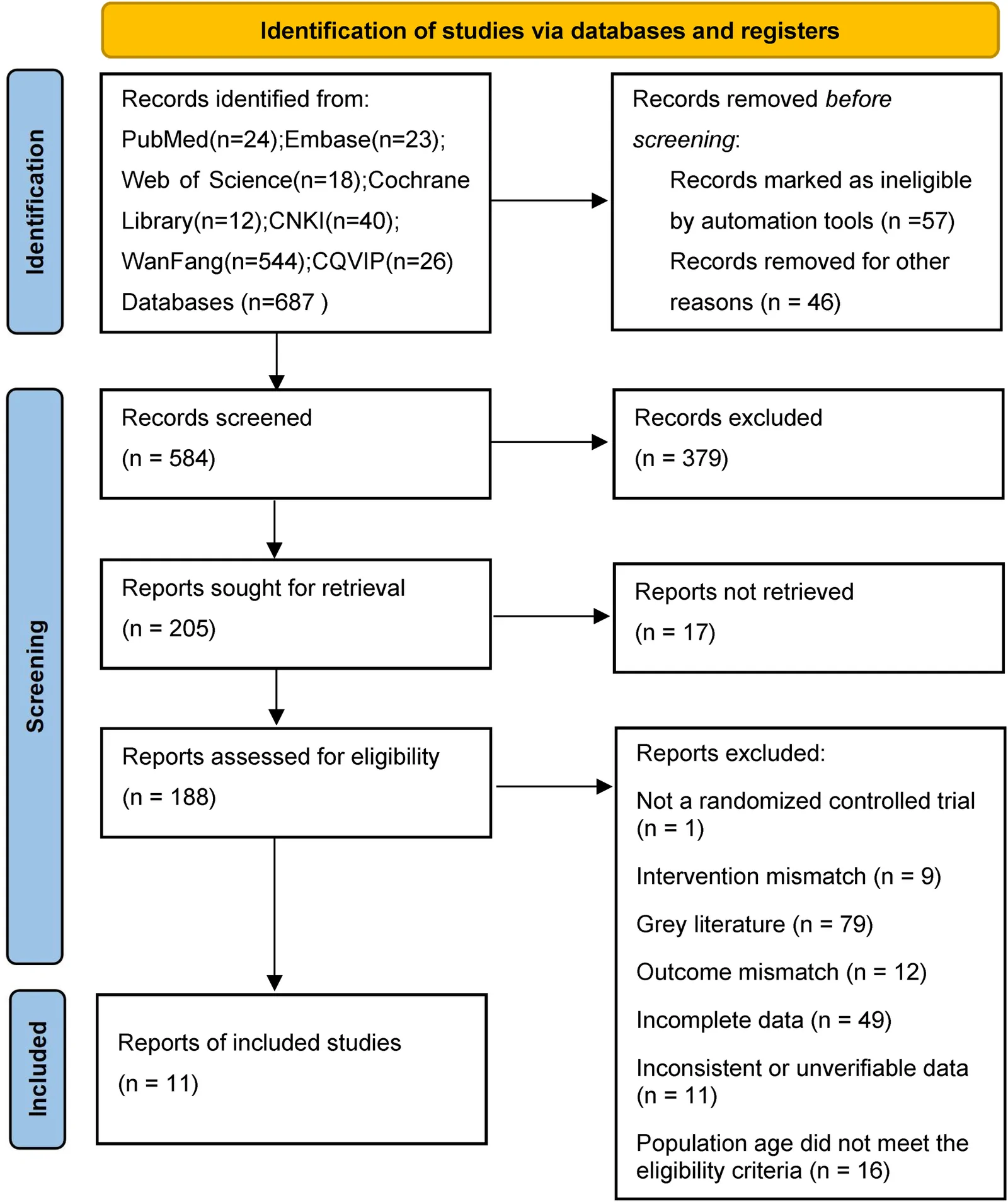
PRISMA flow diagram of study selection.

No language restrictions were imposed during the search, yielding a total of 687 articles in both Chinese and English. After eliminating duplicate records, 584 studies remained. Title and abstract screening excluded 379 studies that were either not RCTs or did not meet the criteria for this analysis, leaving 205 potentially eligible articles. A full-text review, conducted according to predefined inclusion and exclusion criteria, further reduced the selection to 11 studies, as illustrated in Figure 1.

### 3.2 Study characteristics

A total of 4,642 participants from 11 randomized controlled trials were included in this meta-analysis (16, 17, 19–26, 33). All the studies included in this analysis were conducted in China. The majority of participants had chronic heart failure or related HF phenotypes, including HFrEF, systolic HF, chronic congestive HF, elderly CHF, and coronary heart disease–related HF. Most participants were classified as NYHA grading-related clinical responses, classes II–IV or III–IV. The follow-up duration varied widely across studies, ranging from 3 to 24 weeks in most trials, with one multicenter trial reporting a median follow-up of 18.3 months. All studies utilized conventional treatment or guideline-directed medical therapy (GDMT) as the background therapy. The comparator interventions included placebo plus conventional treatment, GDMT alone, or, in one study, sacubitril/valsartan-based therapy alone. In the majority of studies, QLQX was administered orally as four capsules, three times daily. SGLT2 inhibitors were explicitly allowed in only one study, while they were not included in the background regimen of the other studies. See Table 1.

**Table 1:** Basic characteristics of included trials. T/C indicates treatment/control. Natriuretic peptide reporting was extracted exactly as presented in each original trial (endpoint value, change from baseline, percent change, or proportion achieving a prespecified reduction).

Study	Year	Country	Design	Follow-up	HF population	Key inclusion criteria	Age T/C (years)	Male T/C (%)	Sample T/C (n)	Intervention/Comparator	Background CT/GDMT	Baseline LVEF T/C (%)	Key assessment time points	Safety reporting
Cheang	2024	China	Multicenter randomized, double blind, placebo-controlled trial	Median 18.3 months	HFrEF	LVEF ≤40%; NYHA II–III (or IV within 2 weeks before enrollment); NT-proBNP ≥450 pg/mL; stable symptoms	62.56 ± 12.18/ 62.52 ± 12.54	70.42%/ 73.83%	1555/1555	QLQX 4 capsules tid + standard HF therapy vs. Placebo 4 capsules tid + standard HF therapy	ACEI/ARB/ARNI+*β*-blocker + MRA required unless contraindicated/intolerant; SGLT2i allowed	32.20 ± 6.02/32.16 ± 6.08	3 months for NT-proBNP; event-driven follow-up	Yes, AEs/SAEs and discontinuation were reported
Li	2013	China	Multicenter randomized, double-blind, placebo-controlled trial (23 centers)	12 weeks	CHF	CHF ≥3 months; NYHA II–IV; LVEF ≤40%; NT-proBNP ≥450 pg/mL; stable on optimal medical therapy ≥2 weeks	56.98 ± 11.59/ 57.53 ± 11.05	74.59%/ 76.11%	244/247	QLQX 4 capsules tid + CTs vs. Placebo + CTs	Standard HF therapy/usual care per treating physicians	31.85 ± 6.41/ 31.86 ± 6.46	4, 8, and 12 weeks	Yes, AEs/SAEs reported
Zhu	2025	China	Single-center randomized controlled parallel-group trial	8 weeks	CHF	NYHA II–IV; disease duration ≥3 months; BNP ≥35 pg/mL	71.47 ± 4.64/ 72.70 ± 5.25	45.10%/ 48.00%	51/50	QLQX 4 capsules tid + GDMT vs. GDMT alone	GDMT could include diuretics, nitrates, digoxin, ACEI/ARB, and β-blockers	42.63 ± 5.39/ 43.82 ± 6.92	Baseline and 8 weeks	Yes, adverse events were recorded throughout the study
Li	2019	China	Single-center randomized placebo-controlled trial	3 weeks	CHF	symptomatic HF duration >6 months; NYHA II–IV	52.1 ± 3.0/ 51.8 ± 2.8	50.0%/ 57.5%	80/80	QLQX 4 capsules tid + CTs vs. Placebo + CTs	Conventional anti-HF therapy, including cardiotonic and diuretic treatment	37.21 ± 1.51/ 36.23 ± 1.02	Baseline and 3 weeks	Yes; no serious adverse reactions reported
Weng	2019	China	Single-center randomized placebo-controlled trial	12 weeks	CHD with HF	CHD with HF; NYHA II–IV	69.14 ± 6.37/ 69.09 ± 6.14	56.25%/ 58.33%	48/48	QLQX 4 capsules tid + CTs vs. Placebo + CTs	Conventional western therapy, including cardiotonic, diuretic, antihypertensive, coronary-flow–improving, and nitrate drugs	35.13 ± 3. 62/35.19 ± 3.68	Baseline and 12 weeks	Yes; no significant between-group difference in adverse reactions stated
Yang	2017	China	Single-center randomized placebo-controlled trial	24 weeks	CHD with HF	CHD with HF; LVEF <50%; NYHA II–IV; 6MWT 150–425 m	70.24 ± 6.92/70.32 ± 7.05	46.0%/42.0%	50/50	QLQX 4 capsules tid + CTs vs. Placebo + CTs	Digoxin, spironolactone, enalapril, and metoprolol (dose adjusted as needed)	34.86 ± 10.69/34.82 ± 11.26	Baseline and 24 weeks	NR
Chen	2021	China	Single-center randomized controlled trial	4 weeks	CHF	NYHA II–IV	72.3 ± 1.7/72.0 ± 1.5	55.56%/ 59.61%	54/52	QLQX 4 capsules tid + CTs vs. CTs alone	Spironolactone, hydrochlorothiazide, metoprolol, enalapril, and digoxin	35.29 ± 5.82/35.67 ± 5.72	Baseline and 4 weeks	NR
Gao	2017	China	Single-center randomized controlled trial	12 weeks	CHF	CHF; NYHA II–IV; ECG ST–T changes with arrhythmia/conduction block; TCM syndrome reported	61.08 ± 8.41/60.46 ± 8.32	61.36%/ 65.90%	44/44	QLQX 4 capsules tid + CTs vs. CTs alone	Conventional anti-HF therapy, including ACEI/ARB, β-blockers, aldosterone antagonists, and selected vasodilators, digitalis, diuretics, statins	40.94 ± 5.19/41.25 ± 5.31	Baseline and 12 weeks	NR
Liu	2019	China	Single-center randomized controlled trial	8 weeks	CHF	CHF per 2014 guideline; NYHA II–IV; no relevant treatment before admission	65.32 ± 6.32/65.40 ± 6.38	59.57%/55.32%	47/47	QLQX 4 capsules tid + CTs vs. CTs alone	Conventional therapy, including ACEI/ARB, diuretics, β-blockers, and digitalis	30.11 ± 6.32/30.15 ± 6.30	Baseline and 8 weeks	NR
Mao	2013	China	Single-center randomized parallel-group trial	90 days treatment + 30-day follow-up	CHF	informed consent; TCM syndromes of qi deficiency/blood stasis and heart–kidney yang deficiency	65.0 ± 2.0/64.5 ± 2.0	50.0%/ 60.0%	100/100	QLQX 4 capsules tid + CTs vs. CTs alone	Conventional therapy, including digitalis, diuretics, and ACE inhibitors, adjusted as needed	39.2 ± 5.8/41.5 ± 6. 3	Baseline, end of 90-day treatment, and 30-day follow-up	Yes, adverse reactions observed
Wang	2025	China	Single-center randomized controlled trial	8 weeks	CHF	NYHA II–IV; no recent related treatment; dual Western and TCM diagnostic criteria for CHF	60.35 ± 5.41/58.75 ± 5.35	54.17%/47.92%	48/48	QLQX 4 capsules tid + CTs + sacubitril valsartan vs. CTs + sacubitril valsartan	Antiplatelet, antihypertensive, diuretic, vasodilator, glucose-lowering therapy, exercise guidance, sodium restriction, and sacubitril valsartan	36.76 ± 3.64/38.34 ± 4.17	Baseline and 8 weeks	Yes, dizziness, vomiting, and GI reactions monitored; no significant between-group difference

ACEI, angiotensin-converting enzyme inhibitor; AE, adverse event; ARB, angiotensin receptor blocker; ARNI, angiotensin receptor neprilysin inhibitor; BNP, B-type natriuretic peptide; CHF, chronic/congestive heart failure; CHD, coronary heart disease; CTs, conventional therapies; GDMT, guideline-directed medical therapy; HFrEF, heart failure with reduced ejection fraction; MRA, mineralocorticoid receptor antagonist; NR, not reported; NYHA, New York Heart Association; QLQX, QiliQiangxin Capsules; SAE, serious adverse event; SGLT2i, sodium-glucose cotransporter 2 inhibitor.

### 3.3 Risk of bias in studies

Among the 11 included studies, eight detailed their methods for random sequence generation and were judged to be at “low risk” of bias. The remaining three studies (22–24) merely mentioned randomization without specifying the method; thus, they were categorized as “unclear risk.” The reporting rate for allocation concealment was relatively low. Only three studies (16, 17, 26) explicitly reported implementation details and were assessed as “low risk.” The other eight studies failed to mention this process and were accordingly deemed to have an “unclear risk.”

Regarding the blinding of participants and personnel, five studies (19, 21, 24, 26, 33) failed to implement this measure, resulting in a determination of “high risk.” One study (25) did not specify its blinding details and was rated as “unclear risk,” while the remaining five studies successfully implemented double-blinding and were rated as “low risk.”

For the blinding of outcome assessment, six studies were identified as “low risk.” The remaining five (19, 21, 24, 25, 33) did not state their methods and were viewed as “unclear risk.” Notably, Wang (21) was judged to have a “high risk” of selective reporting bias, as their results did not fully cover the predefined measures.

**Figure 2:**
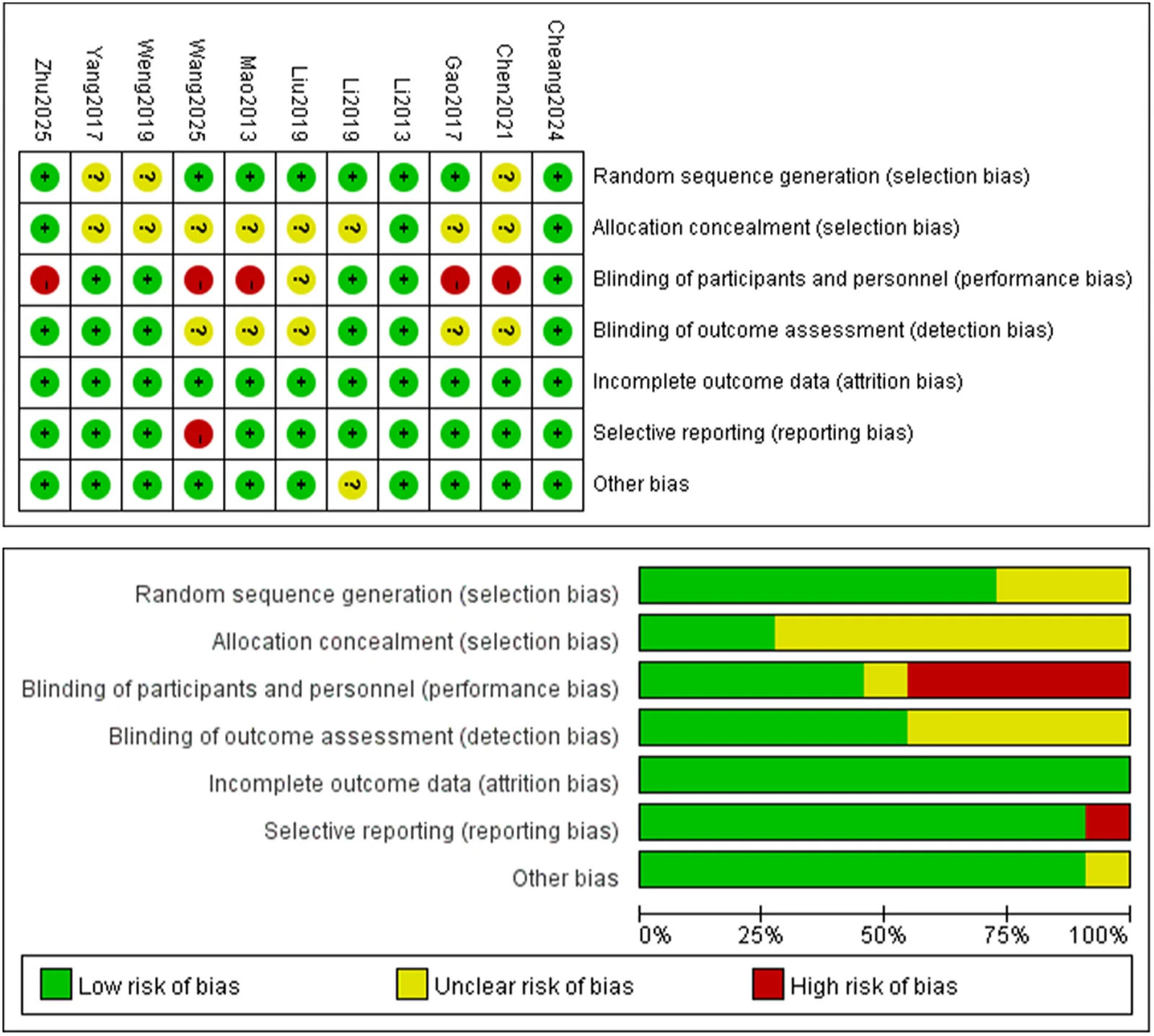
Risk of Bias (RoB) assessment: domain-specific judgments for all included trials.

None of the 11 included studies exhibited a high attrition rate, and missing data had no substantial impact on the outcomes; therefore, all were rated as “low risk.” Overall, the methodological quality of the 11 included RCTs was of a moderate level, with the primary limitations concentrating on the implementation of blinding and allocation concealment, see Figure 2.

### 3.4 Primary outcomes

Heterogeneity was assessed using Cochran's *Q* test and the I² statistic. In accordance with the prespecified statistical analysis framework, continuous primary outcomes were pooled using random-effects models to account for anticipated between-study variability. Prespecified subgroup analyses were conducted according to treatment duration, age category, and study sample size. Sensitivity analyses were performed by sequentially excluding individual studies to assess the robustness of the pooled estimates. When substantial heterogeneity was detected, additional exploratory analyses were conducted to investigate potential sources of heterogeneity.

#### 3.4.1 LVEF

**Figure 3:**
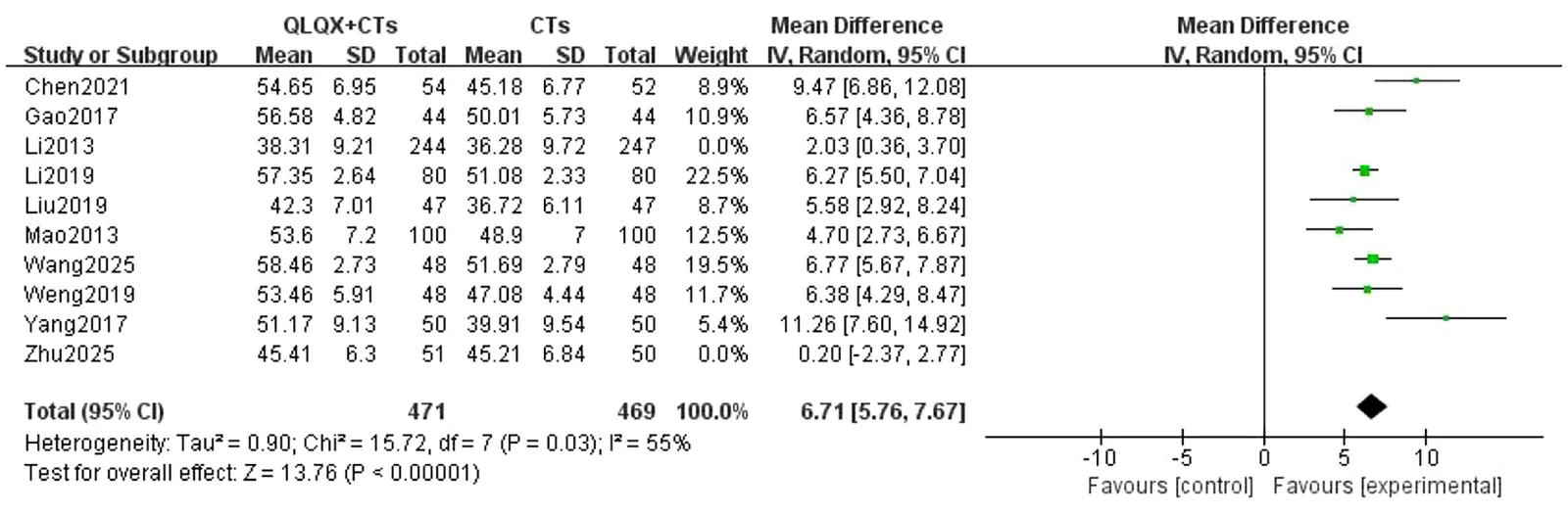
LVEF sensitivity analysis.

**Figure 4:**
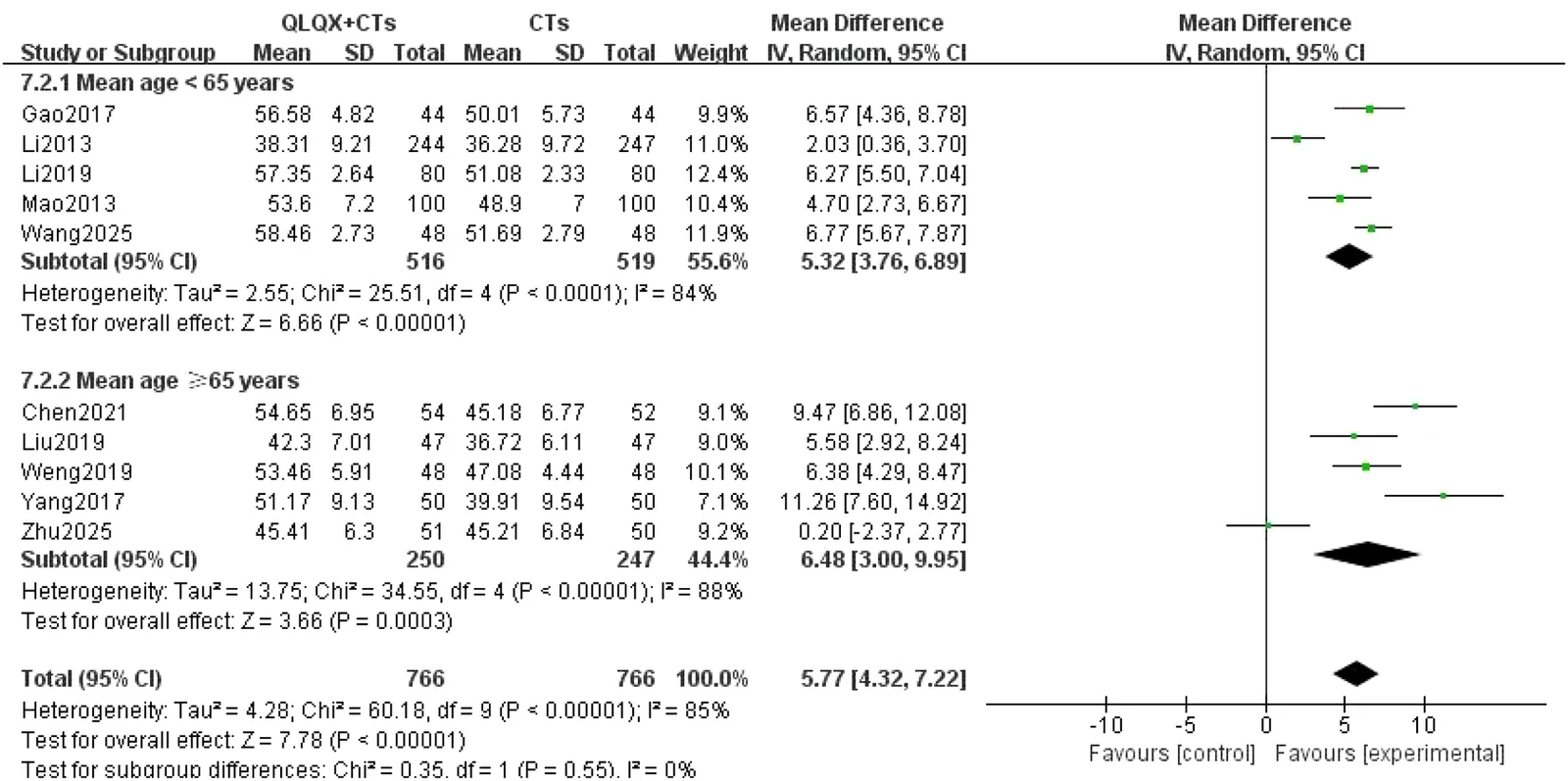
Subgroup analysis by middle-aged and older adults.

LVEF data were available from 10 studies involving 1,532 participants. The large, high-quality QUEST trial by Cheang et al. (16) (*n* = 3,110) did not report LVEF values and therefore was not included in this outcome analysis. In the primary analysis, which retained all studies with available LVEF data, QLQX plus CTs significantly improved LVEF compared with CTs alone [MD = 5.77, 95% CI (4.32, 7.22); *P* < 0. 00001; I² = 85%] (Supplementary File 3, Figure 1). Given the substantial heterogeneity, we performed a leave-one-out sensitivity analysis. Exclusion of the studies by Li et al. (17) and Zhu et al. (26) reduced heterogeneity, while the pooled effect remained significant [MD = 6.71, 95% CI (5.76, 7.67); *I*^2^ = 55%] (Figure 3), supporting the robustness of the overall finding. Age-based subgroup analyses were conducted separately using the full set of studies contributing to the LVEF outcome and showed no significant subgroup difference (Figure 4).

The substantial heterogeneity observed in the LVEF analysis was likely attributable, at least in part, to Li et al. (17) and Zhu et al. (26), which differed from the other included trials in several important respects. Li et al. (17) was a large, multicenter, double-blind, placebo-controlled trial conducted in clinically stable patients with LVEF ≤40% and elevated NT-proBNP who were receiving optimized background therapy, and it reported a comparatively smaller treatment effect on LVEF. In contrast, Zhu et al. (26) enrolled a broader CHF population with a predominance of HFpEF/HFmrEF and a higher baseline LVEF, and no significant between-group differences in echocardiographic parameters were observed after 8 weeks of treatment (Supplementary File 3, Figure 2). These differences in HF phenotype, baseline LVEF, background therapy, and trial design may have contributed to the observed heterogeneity.

Subgroup analyses demonstrated significant LVEF improvements across age groups (<65 years vs. ≥65 years) with no statistically significant difference between subgroups (*P* = 0.55; Figure 4). Exploratory analyses by follow-up duration (≤8 weeks vs. >8 weeks) and study sample size indicated that the overall effect direction remained consistent, although small-sample studies contributed to residual heterogeneity (Supplementary File 3, Figures 2, 3).

Subgroup analysis based on baseline LVEF suggested that QLQX combined with conventional therapy was associated with an improvement in patients with LVEF ≤40% [MD = 6.26, 95% CI (4.81, 7.70); *P* < 0.00001; I² = 83%]. In the LVEF 41%–49% subgroup, the pooled estimate did not show a statistically significant difference between groups [MD = 3.42, 95% CI (−2.82, 9.66); *P* = 0.28; I² = 93%], although the wide confidence interval indicates considerable uncertainty. No statistically significant interaction between subgroups was observed (Chi² = 0.75, *P* = 0.39), and therefore no firm conclusion can be drawn regarding effect modification by baseline LVEF category (Supplementary File 3, Figure 4).

#### 3.4.2 NT-proBNP

The meta-analysis of NT-proBNP included 3,249 patients from seven trials. Among these, two high-quality studies (16, 17) reported only 12-week changes from baseline, whereas all other studies provided post-intervention absolute values. Notably, both high-quality studies presented NT-proBNP data as medians with interquartile ranges (IQRs), reflecting its skewed distribution.

**Figure 5:**
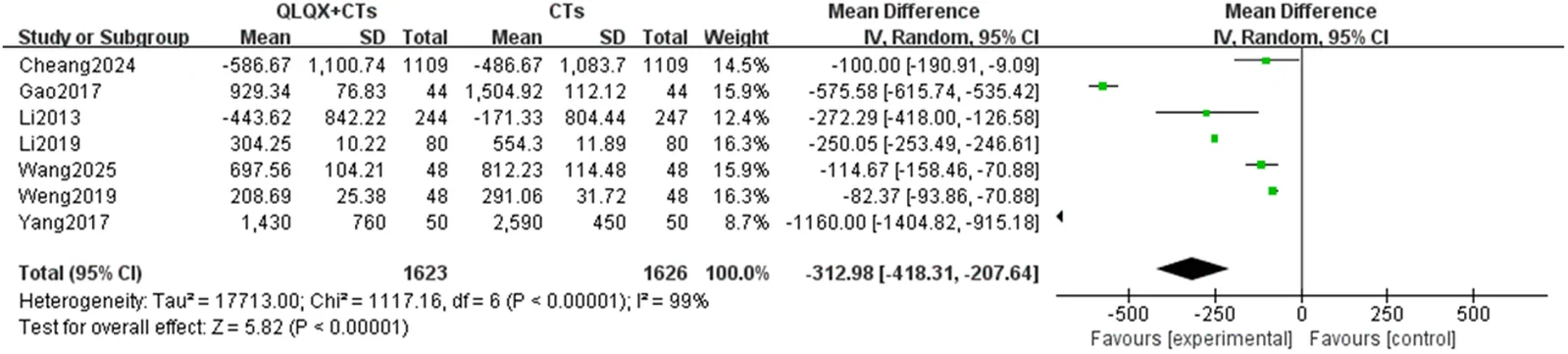
Comparing QLQX plus CTs to CTs alone or placebo plus CTs on the NT-proBNP outcomes.

According to the Cochrane Handbook for Systematic Reviews of Interventions, for continuous outcomes measured on the same scale in randomized studies, change from baseline values and post-intervention values may be combined within a single meta-analysis when the unstandardized MD is used as the effect measure (34). Accordingly, we converted medians and IQRs into SDs using the validated method proposed by Wan et al. (18). We then pooled the transformed change scores and endpoint values using MDs under a random-effects model (Figure 5).

In order to explore the impact of those studies that report NT-proBNP changes in median rather than mean on the results, we conducted a sensitivity analysis and excluded the two studies (16, 17). After excluding the above studies, the aggregate MD = −362.85pg/mL [95% CI (−486.83, −238.87), *P* < 0.00001], and the results were still statistically significant. Excluding these studies did not significantly change the overall conclusion and did not materially alter the direction of effect (Supplementary File 3, Figure 5).

To account for differences in reporting format, we additionally performed a subgroup analysis by data type, categorizing studies into “change score” and “endpoint score” subgroups. Adjunctive QLQX significantly reduced NT-proBNP levels in the overall population [MD = −312.98pg/mL, 95% CI (−418.31, −207.64), *P* < 0.00001] (Figure 5). Importantly, significant reductions in NT-proBNP were observed in both the change score subgroup (MD = −176.35pg/mL, *P* = 0.04) and the endpoint score subgroup (MD = −362.85pg/mL, *P* < 0.00001) (Supplementary File 3, Figure 6). The test for subgroup differences showed no significant interaction according to reporting method (*P* = 0.08), suggesting that pooling these data was acceptable, although the result should be interpreted cautiously given the substantial residual heterogeneity and skewed distribution of NT-proBNP. Nevertheless, overall heterogeneity remained substantial. Neither leave-one-out analysis nor prespecified subgroup analyses based on age, follow-up duration, and sample size identified a clear source of heterogeneity (Supplementary File 3, Figures 7–9).

Several factors may explain this finding. First, baseline NT-proBNP levels varied considerably across studies, suggesting that disease severity at study entry was not uniform. Second, differences in treatment duration, concomitant conventional therapy, and overall treatment context may have influenced the magnitude of NT-proBNP reduction. Third, inconsistencies in follow-up duration, sampling time points, and laboratory measurement conditions may also have contributed to between-study variability. Taken together, although the overall findings support a potential benefit of QLQX plus CTs in improving NT-proBNP levels, the certainty and clinical interpretability of this biomarker finding remain limited. Further high-quality randomized controlled trials with more standardized designs and more consistent outcome reporting are needed to confirm these findings.

#### 3.4.3 LVEDD

The LVEDD analysis included nine studies with 1,332 participants. Compared with CTs alone, QLQX plus CTs was associated with lower LVEDD in predominantly middle-aged and older adults with HF [MD = −3.50, 95% CI (−4.65, −2.35); *P* < 0.00001; I² = 75%] (see Supplementary File 3, Figure 10).

To further investigate the sources of heterogeneity, a leave-one-out sensitivity analysis was conducted. The substantial heterogeneity observed in the LVEDD analysis was likely attributable, at least in part, to Li et al. (17) and Zhu et al. (26). The study by Li et al. (17) was a large, multicenter, double-blind trial conducted in clinically stable patients receiving optimized background therapy, did not show a significant between-group difference in LVEDD after 12 weeks. In contrast, Zhu et al. (26) enrolled a broader CHF population with a predominance of HFpEF/ HFmrEF and relatively preserved baseline systolic function under GDMT. These differences in HF phenotype, baseline cardiac structure, background therapy, and treatment duration may have contributed to the observed heterogeneity. Because Li et al. (17) was a high-quality study, it was retained in the primary analysis.

**Figure 6:**
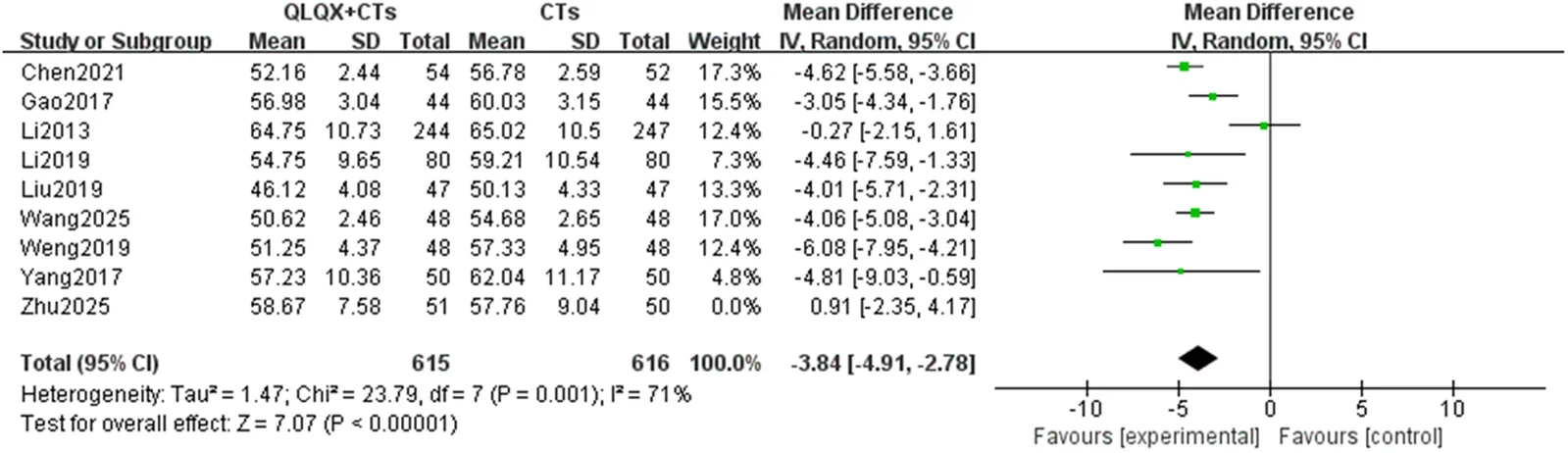
LVEDD sensitivity analysis.

After excluding Zhu et al. (26) the combined result still indicated a significant reduction in LVEDD with the combined treatment [MD = −3.84, 95% CI **(**−4.91, −2.78), *P* < 0. 00001] (Figure 6), with a slight reduction in heterogeneity (I² = 71%), suggesting the direction of effect remained consistent. The sensitivity analysis using the one-by-one exclusion method confirms that after excluding the research that leads to heterogeneity, the overall combined estimate remains stable, which further verifies the robustness of the research results.

Combined treatment in the treatment cycle ≤8 weeks [MD = −3.78, 95% CI (−4.95, −2.61), *P* < 0.00001; I² = 61%] and >8 weeks [MD = −3.41, 95% CI (−5.94, −0.89), *P* = 0.008; I² = 84%] In the two subgroups, the LVEDD was significantly reduced (see Supplementary File 3, Figure 11), and the difference between the two subgroups was not statistically significant (*P* = 0.80). The study-level subgroup analysis by mean age showed that LVEDD decreased both in studies with a mean participant age ≥65 years [MD = −4.03, 95% CI (−5.69, −2.37), *P* < 0.00001; I² = 71%] and in studies with a mean participant age <65 years [MD = −2.91, 95% CI (−4.57, −1.25), *P* = 0.0006; I² = 76%] (Supplementary File 3, Figure 12). The test for subgroup differences was not statistically significant (*P* = 0.35), providing no clear evidence that mean age modified the treatment effect, exploratory subgroup analysis suggested a possible study-level difference, which should be interpreted cautiously. In the sample size of 100 ≤ N < 150 [MD = −2.92, 95% CI (−6.52, 0.68), *P* = 0.11; I² = 81%] or N ≥ 150 [MD = −2.17, 95% CI (−6.26, 1.92), *P* = 0.30; I² = 80%], no significant therapeutic effect was observed (see Supplementary File 3, Figure 13), and the sample size could not explain the heterogeneity between studies (*P* = 0.55).

In summary, existing evidence shows that the combined application of QLQX and CTs can improve ventricular reconstruction in middle-aged and elderly patients and reduce LVEDD. Although the research results have some heterogeneity, the results of the sensitivity analysis and subgroup analysis further confirm the robustness of this conclusion.

### 3.5 Secondary outcomes

#### 3.5.1 NYHA grading-related clinical responses

**Figure 7:**
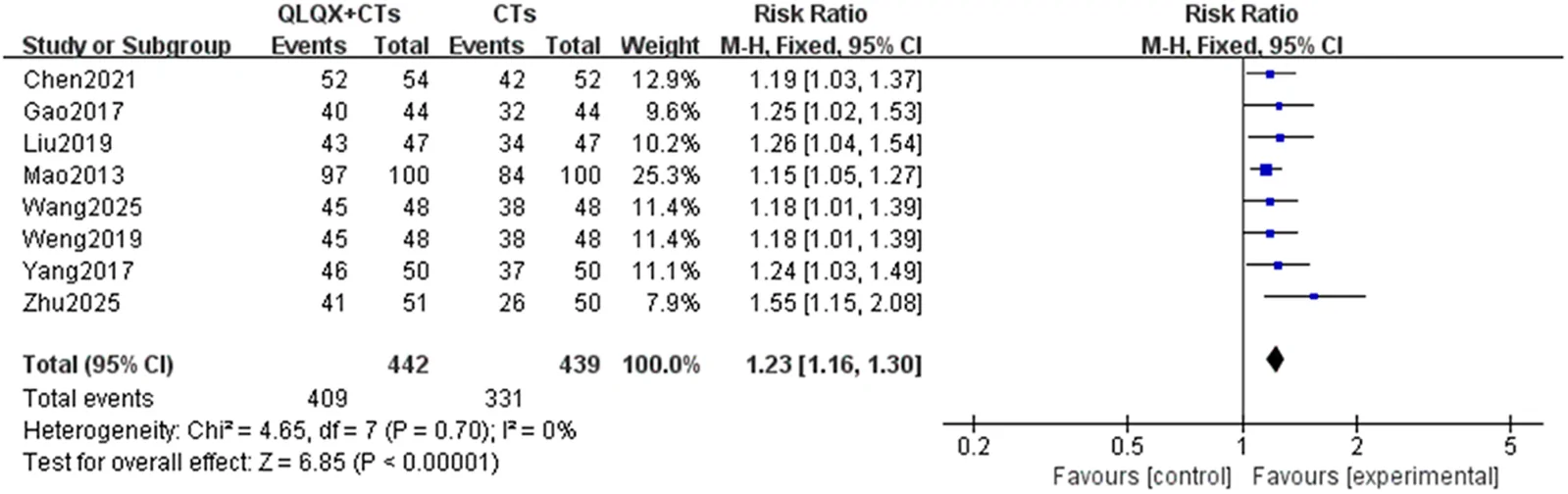
Efficacy analysis of NYHA grading-related clinical response improvement. Tips: improvement was defined as at least one-class improvement or trial-defined clinical response mapped to improvement/non-improvement.

Eight studies reported (*n* = 881) results from the NYHA grading-related clinical response classification using trial-defined clinical response categories, typically including “markedly effective,” “effective,” and “ineffective.” To standardize the data across studies, we reclassified the original trial-defined clinical response categories into a binary outcome: improvement vs. non-improvement. Cases categorized as “markedly effective” or “effective” were grouped as improvement, while those categorized as “ineffective” were classified as non-improvement. A fixed-effect model was applied due to the low statistical heterogeneity between the studies (I² = 0%). The pooled analysis revealed that QLQX combined with CTs was associated with a significantly higher rate of clinical improvement compared to CTs alone [RR = 1.23, 95% CI (1.16, 1.30); Z = 6.85, *P* < 0.00001] (Figure 7). However, this finding should be interpreted with caution, as the harmonized response categories may not have been fully equivalent to a uniform change in NYHA grading-related clinical response classification across all trials. Additionally, several studies used an open-label design, which may have introduced bias in the assessment of subjective outcomes. Consequently, this binary reclassification does not equate to a uniform one-class improvement in NYHA function class, and the results are better interpreted as an overall clinical response rather than a strict functional class change.

#### 3.5.2 6MWD

**Figure 8:**
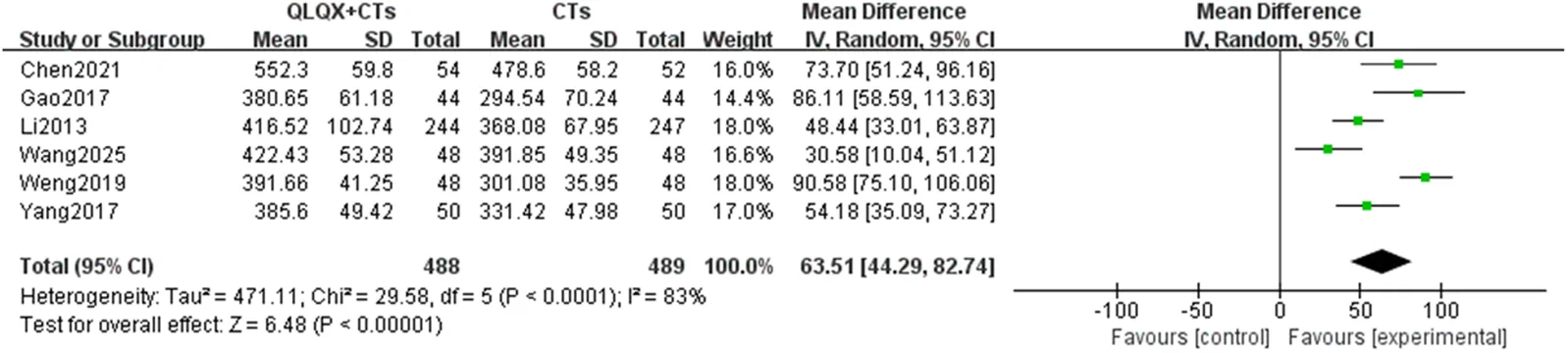
Comparing QLQX plus CTs to CTs alone or placebo plus CTs on the 6MWD outcomes.

In the analysis of the outcome of the 6MWD, a total of 6 studies were included, including 488 participants in the experimental group and 489 participants in the control group. The overall summary effect results show that the combined application of QLQX and CTs significantly improved 6MWD [MD = 63.51, 95% CI (44.29, 82.74), *P* < 0.00001] (Figure 8). Although high heterogeneity (I² = 83%) was observed, no significant source of heterogeneity was found in the sensitivity analysis through the “one-by-one method.” It is worth noting that the effect estimates obtained by all studies tend to be in the experimental group. Although there are significant differences in the magnitude of benefits between studies, the increase of 63.51 meters may still be of clinical significance. However, this result should be interpreted cautiously, especially considering that none of these six trials used a double-blind design, which may introduce a certain degree of subjectivity. In addition, the observed therapeutic effect may be affected by factors such as the severity of the disease, baseline functional status, follow-up duration and patient characteristics. Therefore, in view of the significant heterogeneity between studies, it is more appropriate to interpret this result as a hint of “potential clinical benefits” rather than providing evidence of “definitive evidence of clinically meaningful benefit.” In order to further explore the source of heterogeneity, this study conducted a subgroup analysis based on the duration of follow-up and sample size.

Subgroup analysis based on follow-up duration showed that whether it was short-term follow-up (≤8 weeks) or long-term follow-up (>8 weeks), the experimental group was better than the control group [MD = 51.89, 95% CI [9.64, 94.14], *P* = 0.02; MD = 69.15, 95% CI [46.39, 91.92], *P* < 0.00001] (see Supplementary File 3, Figure 14), although the heterogeneity is still high. The subgroup analysis based on the sample size also showed that in the study with a large sample size, the 6MWD of the experimental group was significantly higher than that of the control group [MD = 56.96, 95% CI (42.98, 70.94), *P* < 0.00001]. In the study with a small sample size, the experimental group also showed significant advantages [MD = 69.00, 95% CI (29.25, 108.75), *P* = 0.0007] (see Supplementary File 3, Figure 15). No statistically significant differences were found between subgroups.

In summary, QLQX combined with CTs may help improve the 6MWD for middle-aged and elderly patients with heart failure, although there is still significant heterogeneity. In view of the lack of double-blind research, the interpretation of the above research results should be cautious.

#### 3.5.3 HF hospitalization or re-admission for HF

Two studies reported HF hospitalization or readmission and were included in this analysis. Cheang et al. (16) reported hospitalization for HF, whereas Li et al. (17) reported readmission for HF as part of a composite cardiac event outcome. Although outcome wording and follow-up duration differed, both measures reflected HF-related hospitalization events and were therefore combined as a clinically relevant endpoint.

**Figure 9:**
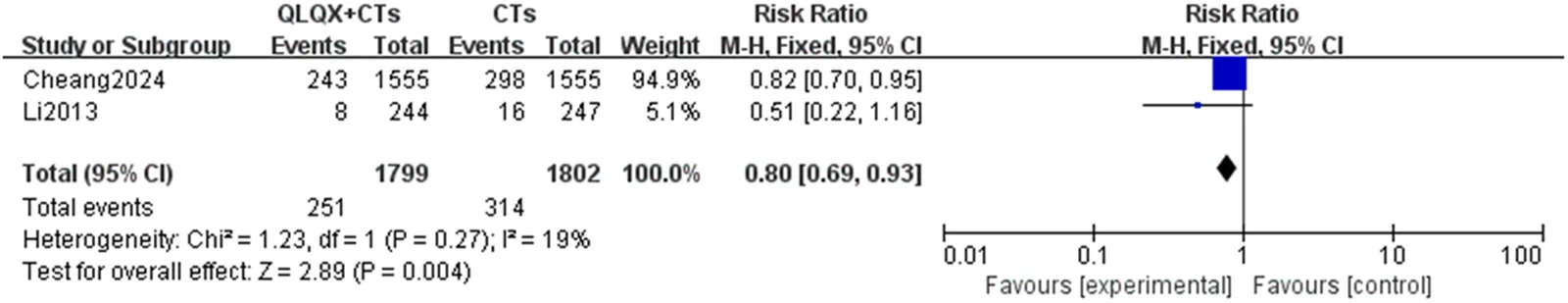
Forest plot of HF hospitalization or re-admission for HF.

Among 1,799 participants receiving QLQX plus CTs, 251 HF hospitalization or readmission events occurred; among 1,802 control participants, 314 events occurred. Because statistical heterogeneity between the two studies was low, a Mantel-Haenszel fixed-effect model was used (Chi² = 1.23, df = 1; *P* = 0.27; I² = 19%). The pooled estimate suggested a lower cumulative risk of HF hospitalization or readmission with QLQX plus CTs [RR = 0.80, 95% CI (0.69, 0.93); Z = 2.89; *P* = 0.004] (Figure 9).

This result should be interpreted cautiously because only two studies contributed data. The pooled estimate was driven mainly by the large trial by Cheang et al. (16) which contributed 94.9% of the statistical weight, whereas Li et al. (17) contributed 5.1%. The finding therefore suggests a lower cumulative risk of HF-related hospitalization within the included follow-up periods, not a pooled time-to-event effect.

#### 3.5.4 Publication bias

Publication bias was assessed using funnel plots for LVEF, NT-proBNP, LVEDD, 6MWD, and NYHA-related clinical response and improvement outcomes (Supplementary File 3, Figure 16). Although no obvious asymmetry was observed, visual inspection alone is insufficient to draw definitive conclusions. For LVEF, which included 10 studies, a formal test for funnel plot asymmetry was additionally performed. Egger's regression test did not identify statistically significant funnel plot asymmetry for LVEF [Egger's test: intercept = −0.46, 95% CI (−4.79, 3.87), *P* = 0.812] (Supplementary File 3, Figures 17, 18). For NT-proBNP, LVEDD, 6MWD, NYHA-related clinical response, and HF hospitalization or readmission, formal statistical tests were not conducted because each outcome included fewer than 10 studies. Therefore, although no apparent funnel plot asymmetry was observed visually, publication bias and small-study effects cannot be completely excluded.

#### 3.5.5 GRADE assessment

The evidence certainty of all preset outcome indicators is evaluated using the GRADE method, covering LVEF, NT-proBNP, NYHA grading-related clinical responses, LVEDD, 6MWD, and HF hospitalization or rehospitalization. Overall, the certainty of the evidence is between medium and extremely low (see Supplementary File 4 for details).

The main reasons for the downgrade of evidence include bias risk, inconsistency, indirectness and inaccuracy. There are many methodological limitations in the included studies, which are manifested in the unclear process of random sequence generation, insufficient distribution of hidden reports, and the failure of subjects, researchers, or outcome evaluators to implement the complete blind method. These limitations increase the potential risk of selection bias, implementation bias, and detection bias, which is particularly prominent for outcome indicators that are subjective or dependent on the performance of subjects (such as NYHA heart function grading and 6MWD).

The evidence certainty of LVEF was rated as “medium” and downgraded by one level due to the risk of bias. The evidence certainty of LVEDD was rated as “low,” mainly due to the limitations of methodology and significant heterogeneity between studies. The evidence certainty of NT-proBNP and 6MWD has been rated as “extremely low,” although sensitivity analysis and subgroup analysis have been carried out, serious bias risk and inexplicable heterogeneity still limit our confidence in these outcome indicators.

For NYHA heart function grading, the evidence certainty was rated as “low.” Although the summary results suggest that QLQX combined with CTs can improve clinical response, most trials report their own defined comprehensive efficacy categories, rather than standardized NYHA heart function grading changes. Therefore, the outcome indicator reflects the clinical response defined by each experiment itself, rather than the unified improvement of cardiac function grading. In view of the risk and indirectness of bias, the evidence level of the outcome indicator has been lowered.

For the outcome indicator of HF hospitalization or re-hospitalization due to HF, the evidence certainty was rated as “low.” There are two studies on this outcome indicator. The study of Cheang et al. (16) reported the first hospitalization due to HF, while the study of Li et al. (17) reported the re-hospitalization due to HF as an integral part of the comprehensive heart event. The summary analysis results suggest that the cumulative risk of the outcome indicator has been reduced. However, due to the inconsistent definition of the outcome indicator in each study, the follow-up cycle is different; coupled with the limited number of studies included, the outcome indicator is indirect and inaccurate. It is worth noting that the estimates obtained from this summary analysis are largely influenced by the lead of the experiment carried out by Cheang and others. Therefore, the final evidence level is judged as “low.”

In summary, the existing evidence shows that the QLQX combined CTs treatment regimen may provide additional benefits for patients with heart failure in terms of heart function, functional status and specific clinical outcomes. However, the overall certainty of the evidence is still limited. The findings of this study are limited by factors such as methodological defects, residual heterogeneity, insufficient hard endpoint reporting, and incomplete representation of standardized treatment under the guidance of the current guidelines. Therefore, further large-scale, multi-center, strictly blind randomized controlled trials need to be carried out, and standardized outcome definitions and longer follow-up periods are needed to confirm the above results.

## 4 Discussion

### 4.1 Main findings

In this meta-analysis, middle-aged and older adults were defined as study populations with a mean or median age of ≥45 years, in line with the classification by Shinan-Altman and Werner (13), where middle age is defined as 44–64 years and older age as ≥65 years. A review of cardiovascular disease literature further supports this definition, with middle age commonly starting at 45 years. This age criterion applies at the study level, rather than as a strict individual age cutoff, since most trials reported only the mean or median age, without providing full age distributions. Therefore, the findings pertain to study populations predominantly composed of middle-aged and older adults, rather than strictly age-restricted cohorts.

This meta-analysis suggests that QLQX combined with CTs may be associated with favorable short- to medium-term changes in several surrogate or functional outcomes, including LVEF, NT-proBNP, LVEDD, NYHA-related clinical response, and 6MWD. Limited evidence from two studies also suggests that QLQX combined with CTs may reduce the risk of HF hospitalization or rehospitalization. However, both the number of contributing trials and the number of clinical events were small, and the overall certainty of evidence for this outcome ranged from low to very low. These findings should therefore be interpreted cautiously and regarded as exploratory and hypothesis-generating, rather than as definitive evidence of prognostic benefit.

The main contribution of this review is not to establish a definitive therapeutic benefit of QLQX, but to characterize the current evidence profile in HF study populations composed predominantly of middle-aged and older adults. Overall certainty of evidence ranged from moderate to very low, and the findings were limited by methodological concerns, residual heterogeneity, substantial variation in follow-up duration, limited reporting of hard endpoints, and insufficient representation of contemporary GDMT. Accordingly, these results should be interpreted as preliminary signals that require validation in trials conducted under modern standard-of-care conditions.

### 4.2 Comparison with the previous meta-analyses and QUEST trial

Previous meta-analyses have shown that QLQX, as an adjunct to standard therapy, can improve cardiac function, natriuretic peptide levels, exercise tolerance, and some clinical outcomes in patients with heart failure. Some studies have also focused on hard endpoints including cardiovascular mortality and heart failure-related hospitalizations.

For example, a recent systematic review by Li et al. (9) specifically evaluated the use of QLQX in patients with heart failure with HF-related ejection fraction HFrEF and reported its benefits in cardiovascular death, heart failure hospitalization, composite cardiovascular events, and multiple cardiac function outcomes. However, previous reviews primarily focused on heart failure phenotypes rather than age-related clinical applicability and did not employ a pre-defined framework, focusing mainly on studies involving middle-aged and elderly patients. In contrast, this systematic review uses age criteria at the research level, selecting trials primarily involving middle-aged and elderly heart failure patients, and updates the evidence to January 2026. It also more cautiously synthesizes and analyzes factors such as heart failure phenotype, baseline LVEF, background treatment, follow-up time, heterogeneity in outcome reporting, and GRADE-based evidence certainty. This method allows us to more clearly define the strengths and limitations of existing evidence. Importantly, although the results of the summary are consistent with the early evidence, our interpretation is more conservative because some of the results are affected by high heterogeneity and methodological defects.

Therefore, the added value of this study lies not in replicating previously established efficacy signals, but in refining the interpretation of existing evidence in a more clinically relevant population context. It clarifies the applicability, limitations, and certainty of QLQX in predominantly middle-aged and elderly patients with heart failure, rather than providing further general confirmation of efficacy for HFrEF.

The QUEST trial is an important high-quality randomized, double-blind, placebo-controlled trial aimed at evaluating the QLQX of HFrEF patients Cheang et al. (16). The trial provides relatively strong evidence of clinical outcomes and changes in NT-proBNP, and makes a significant contribution to the current evidence base. However, the QUEST trial does not report the LVEF value, so it cannot be included in the summary analysis of LVEF. In addition, the overall evidence base remains unbalanced because most of the other studies included are small in scale, shorter in time, and less rigorous in the blinding method. Therefore, although the QUEST test enhances the credibility of QLQX as a potential adjuvant treatment, it does not completely address the uncertainty associated with long-term efficacy, applicability to different heart failure phenotypes, and fully optimized incremental benefits under contemporary GDMT.

### 4.3 Clinical interpretation of the findings

The observed increases in LVEF and reductions in LVEDD suggest that QLQX may be associated with modest beneficial effects on cardiac contractility and ventricular remodeling. Sensitivity analyses indicate that, despite heterogeneity, the direction of effect for both LVEF and LVEDD is generally consistent. This heterogeneity may partly reflect differences in heart failure phenotype, baseline cardiac function, background therapy, study design, and treatment duration. For example, some studies enrolled clinically stable HFrEF patients receiving optimized background therapy, whereas others included broader CHF populations with relatively preserved baseline contractility. These factors may influence the magnitude of the observed treatment effect.

NT-proBNP is an important biomarker of heart failure severity and prognosis. In this meta-analysis, QLQX combined with CTs was associated with lower NT-proBNP levels. This finding should, however, be interpreted cautiously. First, NT-proBNP data are typically skewed, and the two high-quality studies reported medians with interquartile ranges, requiring statistical transformation before pooling. Second, some studies reported changes from baseline, while others reported post-treatment endpoint values. Although all data were analyzed using mean differences on the same scale, the mixed reporting formats may introduce uncertainty. Third, substantial residual heterogeneity persisted despite subgroup and sensitivity analyses. Differences in baseline disease severity, treatment duration, concomitant therapy, sampling timing, and laboratory measurement conditions may all influence the pooled estimate.

NYHA functional class and 6MWD are clinically meaningful indicators of symptom burden and functional capacity. The present analysis suggests that QLQX may be associated with improvements in trial-defined NYHA-related clinical response and potential increases in 6MWD. These findings should be interpreted with caution. For NYHA-related outcomes, most trials reported trial-defined response categories, such as “markedly effective”, “effective” and “ineffective”, rather than standardized changes in NYHA functional class. Therefore, the pooled result reflects an overall trial-defined clinical response rather than a uniform one-class improvement in NYHA functional status.

For HF hospitalization or re-admission, evidence was limited to two studies. The pooled analysis suggested a potential reduction in cumulative HF-related hospitalization events with QLQX combined with CTs. However, the small number of studies and differences in outcome definitions and follow-up durations limit the reliability of these findings. Consequently, these results should be considered preliminary and hypothesis-generating rather than conclusive evidence of long-term prognostic benefit. Further large-scale, multicenter, double-blind randomized controlled trials with standardized outcome definitions and sufficient follow-up are needed to confirm these observations.

### 4.4 Applicability to contemporary guideline-directed care in older adults

The applicability of the current findings to contemporary HF care remains uncertain. Most included trials used conventional therapy as background treatment, but these regimens did not consistently reflect modern GDMT. In particular, routine use of ARNI and SGLT2 inhibitors was limited or unclearly reported in most studies. Only one study explicitly allowed SGLT2 inhibitor use, and many earlier trials were conducted before these drugs became core components of contemporary HF management.

This limitation is clinically important because contemporary HF management increasingly relies on multidrug regimens that include ARNI or ACEI/ARB, beta-blockers, MRAs, and SGLT2 inhibitors when indicated. The incremental effect of QLQX may differ when added to fully optimized GDMT rather than to older or lower-intensity background therapy. The pooled estimates should therefore be interpreted as potential adjunctive effects within the treatment contexts represented by the included trials, not as definitive evidence of benefit beyond optimized modern GDMT.

Generalizability may also be limited because all included studies were conducted in China. Differences in ethnicity, healthcare systems, diagnostic practice, background medication patterns, and acceptance of TCM may affect external validity. International or multicenter trials conducted under contemporary GDMT conditions are needed to determine whether the observed associations extend to broader HF populations.

### 4.5 Mechanistic plausibility

From the perspective of the mechanism of action, the clinical benefits shown by QLQX have a reasonable biological explanation. The pathological characteristics of heart failure include oxidative stress, excessive inflammatory response, myocardial energy metabolism disorders, and continuous activation of cell death pathways. The main components of QLQX (including astragalus and ginseng) are reported to improve heart function through the synergy of multiple targets and multiple pathways. Its specific mechanisms cover many aspects, such as inhibiting ventricular reconstruction, reducing oxidative stress, and alleviating myocardial fibrosis (35). Experimental studies further suggest that QLQX may reduce cardiomyocyte apoptosis via the ROS/AMPK/mTOR pathway (36) and improve myocardial energy metabolism by upregulating HIF-1*α* and downstream glycolytic enzymes, thereby enhancing substrate utilization and ATP production (37). These mechanisms may help explain the improvements observed in LVEF, NT-proBNP, and LVEDD. Nevertheless, these experimental findings should be regarded as supportive rather than confirmatory, because the present meta-analysis was not designed to establish causal mechanistic links in humans.

## 5 Strengths and limitations

This review has many advantages. The research process strictly follows the PRISMA 2020 guidelines and only incorporates randomized controlled trials, thus effectively improving the internal validity of the evidence. This review focuses on middle-aged and older adults with HF as the main body, and comprehensively evaluates multiple therapeutic effect dimensions such as heart function, ventricular reconstruction, symptoms, exercise tolerance, and specific clinical outcomes. The researchers pre-set and implemented subgroup analysis and “leave-one-out” sensitivity analysis to assess the heterogeneity and robustness of the research results. At the same time, this review also adopts the GRADE grading method to enhance the transparency of the evidence interpretation process.

However, there are also some limitations worth looking at in this review. The quality of the methodology included in many experiments still needs to be improved, especially in the lack of allocation concealment and blind implementation, which may increase the risk of selection bias, implementation bias and detection bias. For a number of key continuity outcome indicators, such as NT-proBNP, LVEDD, and 6MWD, the study results showed significant heterogeneity. In addition, there are inconsistencies in the definition of outcome indicators and the reporting methods of each included study. Specifically, the summary analysis of NT-proBNP faces the challenge of multiple complex factors: the data distribution of the indicator is biased; some studies report the change value with the endpoint value; and in two high-quality trials, researchers had to convert the data based on the median report into estimates. The mean and standard deviation are processed. For NYHA grading-related outcome indicators, each trial often only reports its own defined composite response categories, rather than using standardized NYHA grading conversion, which limits the comparability between research results to a certain extent. In addition, some of the experiments adopted an open-label design, which may lead to an overestimation of the effect of some subjective outcome indicators.

Although formal Egger's regression tests did not indicate significant funnel plot asymmetry for the LVEF outcome, this finding should be interpreted cautiously because only 10 studies were available and substantial between-study heterogeneity was present. For the remaining outcomes, the number of included studies was insufficient for reliable formal testing of funnel plot asymmetry. Therefore, the possibility of publication bias or small-study effects cannot be ruled out.

Moreover, most studies had relatively short follow-up periods, and only two reported HF hospitalization or re-admission for HF. This limits the strength of conclusions regarding long-term prognosis and hard clinical endpoints.

Another key limitation is that the target population was defined at the study level rather than at the individual-participant level. Most included trials reported only the mean or median age, or a general description of the study population's age profile, rather than precise participant-level age distributions. Therefore, the eligibility criteria identified studies with populations predominantly composed of middle-aged and older adults, rather than studies that strictly excluded all participants younger than 45 years. Accordingly, the findings should be interpreted as applicable to study populations predominantly composed of middle-aged and older adults, rather than to strictly age-restricted or individually age-stratified populations.

In addition, grey literature, such as theses and conference proceedings, was not included in this analysis. This exclusion may have limited the comprehensiveness of the review by omitting potentially relevant unpublished studies.

Finally, most trials were conducted in Chinese populations, and many were single-center or domestic multicenter studies. In addition, background treatment regimens did not fully reflect contemporary guideline-directed medical therapy. These factors may limit the generalizability and applicability of the findings to current clinical practice. Future studies should adopt adequately powered, multicenter, randomized designs, preferably with double blinding, standardized outcome definitions, consistent reporting procedures, and longer follow-up periods. The effects of QLQX should also be further evaluated based on contemporary guideline-directed medical therapy. Such studies are needed to clarify the long-term efficacy and safety of QLQX, its potential prognostic implications, and the patient subgroups most likely to benefit.

## 6 Conclusion

In predominantly middle-aged and older HF study populations, QLQX plus conventional therapy may be associated with short- to medium-term improvements in surrogate and functional outcomes, including higher LVEF, lower LVEDD, lower NT-proBNP, and improved trial-defined clinical response. These findings suggest potential physiological and functional effects, but they do not establish long-term prognostic benefit. Evidence for hard clinical endpoints remains limited.

Because of methodological limitations, between-study heterogeneity, variable follow-up duration, and differences in background therapy, certainty of evidence ranged from moderate to very low. Future studies should use adequately powered, multicenter, double-blind randomized designs with longer follow-up and standardized outcome definitions. The present findings should be interpreted within the study-level age criteria and treatment contexts represented by the included trials.

Moreover, the incremental value of QLQX in patients receiving fully optimized contemporary GDMT, including ARNI- and SGLT2 inhibitor-containing regimens, remains uncertain and requires further investigation.

## Data Availability

The original contributions presented in the study are included in the article/Supplementary Material, further inquiries can be directed to the corresponding author/s.
