# Supplementary material for: Adjunctive qiliqiangxin capsule for heart failure in predominantly middle-aged and older adults: a systematic review and meta-analysis of randomized controlled trials
Source: Front Cardiovasc Med. 2026 Aug 12;13:1881321. doi: 10.3389/fcvm.2026.1881321 (PMC13506899; doi:10.3389/fcvm.2026.1881321)
Supplement: Supplementary file 1 [file Supplementaryfile4.pdf]

Author(s):
Question: □□□□□ compared to placebo for heart failure
Setting:
Bibliography: . QLQX+CTs versus CTs for heart failure. Cochrane□□□□□□□ [□□]□□[□□].

Certainty assessment							№ of patients		Effect		Certainty	Importance
№ of studies	Study design	Risk of bias	Inconsistency	Indirectness	Imprecision	Other considerations	□□□□□	placebo	Relative (95% CI)	Absolute (95% CI)		
**NYHA function class**												
8	randomised trials	serious[a]	not serious	serious[a]	not serious	none	409/442 (92.5%)	331/439 (75.4%)	**RR 1.23** (1.16 to 1.30)	**173 more per 1,000** (from 121 more to 226 more)	⊕⊕◯◯ Low[a]	
**LVEF**												
10	randomised trials	serious[b]	not serious	not serious	not serious	none	766	766	-	MD **5.77 higher** (4.32 higher to 7.22 higher)	⊕⊕⊕◯ Moderate[b]	
**LVEDD**												
9	randomised trials	serious[c]	serious[c]	not serious	not serious	none	615	616	-	MD **3.84 lower** (4.91 lower to 2.78 lower)	⊕⊕◯◯ Low[c]	
**6MWD**												
6	randomised trials	serious[d]	very serious[d]	not serious	not serious	publication bias strongly suspected	488	489	-	MD **63.51 higher** (44.29 higher to 82.74 higher)	⊕◯◯◯ Very low[d]	
**NT-pro BNP**												
7	randomised trials	serious[e]	very serious[e]	serious[e,f]	not serious	publication bias strongly suspected	270	270	-	MD **362.85 lower** (486.83 lower to 238.87 lower)	⊕◯◯◯ Very low[e,f]	
**HF hospitalization or re-admission for HF**												
2	randomised trials	not serious	not serious	serious[g]	serious[f]	none	251/1799 (14.0%)	314/1802 (17.4%)	**RR 0.80** (0.69 to 0.93)	**35 fewer per 1,000** (from 54 fewer to 12 fewer)	⊕⊕◯◯ Low[f,g]	

CI: confidence interval; **MD:** mean difference; **RR:** risk ratio

Explanations

a. Downgraded by one level for risk of bias because several trials had unclear allocation concealment and blinding; downgraded by one level for indirectness because the endpoint was based on trial-defined clinical response categories rather than standardized NYHA class changes.
b. Downgraded by one level for risk of bias because some included trials has unclear reporting of allocation concealment, blinding, and outcome assessment procedures.
c. Downgraded by one level for risk of bias and one level for inconsistency because between-study heterogeneity was present and could not be fully explained.
d. Downgraded by one level for risk of bias because 6MWD is performance-dependent and may be affected by incomplete blinding; downgraded by two levels for serious unexplained heterogeneity across studies.
e. Downgraded by one level for risk of bias, two levels for serious unexplained heterogeneity, and one level for indirectness because NT-proBNP is a surrogate biomarker rather than a direct patient-important outcome.
f. NT-proBNP is a surrogate biomarker, rather than a patient-perceived outcome or a hard prognostic endpoint. While it reflects the burden of heart failure and prognostic risk, it cannot fully substitute for clinical outcomes such as mortality, heart failure hospitalization, or quality of life. Therefore, due to its indirect nature, it is recommended that its evidentiary weight be downgraded.
g. Downgraded by one level for indirectness because first hospitalization for HF and re-admission for HF were combined, and follow-up duration differed between trials. Downgraded by one level for imprecision because only two studies contributed to the estimate and the pooled effect was largely driven by one large trial.
