# Supplementary material for: Adjunctive qiliqiangxin capsule for heart failure in predominantly middle-aged and older adults: a systematic review and meta-analysis of randomized controlled trials
Source: Front Cardiovasc Med. 2026 Aug 12;13:1881321. doi: 10.3389/fcvm.2026.1881321 (PMC13506899; doi:10.3389/fcvm.2026.1881321)
Supplement: Supplementary file 3 [file Supplementaryfile3.docx]

**Supplementary File 3**

**LVEF**

**Supplementary Figure 1** Comparing QLQX plus CTs to CTs alone or placebo plus CTs on the LVEF outcomes.

**Supplementary Figure 2** Subgroup analysis by treatment duration.

**Supplementary Figure 3** Sample Size Subgroup Analysis.

**Supplementary Figure 4** Effect of QLQX on LVEF stratified by baseline LVEF.

**NT-proBNP**

**Supplementary Figure 5** Sensitivity analysis.

**Supplementary Figure 6** Comparing QLQX plus CTs to CTs alone or placebo plus CTs on the NT-proBNP outcomes.

**Tips:** The two data types were combined using the MD on the same scale and subjected to a subgroup analysis in a reporting-format.

**Supplementary Figure 7** Subgroup analysis by treatment duration.

**Supplementary Figure 8** Age subgroup Analysis.

**Supplementary Figure 9** Sample Size Subgroup Analysis.

**LVEDD**

**Supplementary Figure 10** Comparing QLQX plus CTs to CTs alone or placebo plus CTs on the LVEDD outcomes.

**Supplementary Figure 11** Subgroup analysis by treatment duration.

**Supplementary Figure 12** Age subgroup Analysis.

**Supplementary Figure 13** Sample Size Subgroup Analysis.

**6MWD**

**Supplementary Figure 14** Subgroup analysis by treatment duration.

**Supplementary Figure 15** Sample Size Subgroup Analysis.

**Supplementary Figure 16** Funnel plots of publication bias for each outcome: A: LVEF, B: NT-proBNP, C: LVEDD, D: 6MWD, E: NYHA grading-related clinical response.

**Supplementary Figure 17** Egger’s regression test for funnel plot asymmetry in LVEF.

**Supplementary Figure 18** Results of Egger’s regression test for funnel plot asymmetry in the LVEF outcome.
