# Supplementary material for: Adjunctive qiliqiangxin capsule for heart failure in predominantly middle-aged and older adults: a systematic review and meta-analysis of randomized controlled trials
Source: Front Cardiovasc Med. 2026 Aug 12;13:1881321. doi: 10.3389/fcvm.2026.1881321 (PMC13506899; doi:10.3389/fcvm.2026.1881321)
Supplement: Supplementary file 4 [file Supplementaryfile2.doc]

**Supplementary File 2 Search strategies**

PubMed

#1："(Heart failure[Title/Abstract]) OR (Cardiac failure[Title/Abstract])) OR (CHF[Title/Abstract])

#2：(Qili Qiangxin[Title/Abstract]) OR (Qiliqiangxin[Title/Abstract])) OR (Qili Capsule[Title/Abstract])

#3：(Randomized Controlled Trial[Publication Type]) OR (Randomized[Title/Abstract])) OR (Placebo[Title/Abstract])

#1 AND #2 AND #3

**Embase**

#1：'heart failure'/exp OR 'heart failure':ab,ti OR 'cardiac backward failure':ab,ti OR 'cardiac decompensation':ab,ti OR 'cardiac failure':ab,ti OR 'cardiac incompetence':ab,ti

#2:'qiliqiangxin':ab,ti OR 'qiliqiangxin capsule':ab,ti OR 'qili capsule':ab,ti

#3:'randomized controlled trial'/exp' OR randomized controlled trial':ab,ti OR placebo:ab,ti OR randomized:ab,ti

#1 AND #2 AND #3

**Cochrane Library**

#1：MeSH descriptor: [Heart Failure] explode all trees

#2:(Heart Failure):ti,ab,kw OR (Cardiac failure):ti,ab,kw OR (Congestive Heart Failure):ti,ab,kw OR (cardiac incompetence):ti,ab,kw

#3:#1 OR #2

#4:MeSH descriptor: [Randomized Controlled Trial] explode all trees

#5:(randomized controlled trial):pt OR (placebo):ti,ab,kw OR (randomized):ti,ab,kw

#6:#4 OR #5

#7:（qiliqiangxin）:ti,ab,kw OR (qiliqiangxin capsule):ti,ab,kw OR( 'qili capsule):ti,ab,kw

#8:#3 AND #6 AND #7

**Web of Science**

#1:Heart Failure OR Cardiac failure OR Congestive Heart Failure OR cardiac incompetence (Topic)

#2:randomized controlled trial OR placebo OR randomized(Topic)

#3:qiliqiangxin OR qiliqiangxin capsule OR qili capsule （Topic）

#1AND #2 AND #3

**CNKI**

（主题：心力衰竭） OR（篇关摘：心力衰竭+心衰+心功能不全（精确））AND（主题：芪苈强心）AND（主题：随机对照）OR（篇关摘：随机对照试验 + 随机对照研究）

CQVIP Chinese Journals Platform

(M=芪苈强心) AND (M=心力衰竭 OR M=心衰 OR M=心功能不全) AND (M=随机 OR M=对照)

WanFang

(题名或关键词:("芪苈强心")) AND (题名或关键词:("心力衰竭" OR "心衰" OR "心功能不全")) AND (题名或关键词:("随机" OR "对照"))
